# Heat of Formation of Titanium Trichloride

**DOI:** 10.6028/jres.064A.051

**Published:** 1960-12-01

**Authors:** Walter H. Johnson, Alexis A. Gilliland, Edward J. Prosen

## Abstract

A calorimetric comparison of the heat of hydrolysis of TiCl_4_(liq) with the heat of oxidation and hydrolysis of TiCl_3_(c) has been made. The following value is reported for the combination of these data according to the process:

$$\begin{array}{l} {\text{TiCl}}_{3}\left(c\right)+½I_{2}\left(c\right)+\text{HCl}\left(g\right)\to {\text{TiCl}}_{4}\left(\text{liq}\right)+\text{HI}\left(g\right),\\ \;\Delta H{}^{\circ}\left(25\;{}^{\circ}C\right)=8.37\pm 0.30\;\text{kcal}/\text{mole}. \end{array}$$

A combination of this value with −192.3 ±0.7 kcal/mole for the heat of formation of TiCl_4_(liq) and with the standard heats of formation of HCl(g) and HI(g) gives for TiCl_3_(c), ΔHf°(25 °C) = −172.4±0.8 kcal/mole.

## 1. Introduction

Several independent determinations of the heat of formation of titanium trichloride have been reported during the past few years [1, 2, 3, 4, 5, 6].^1^ The only values previously available were those estimated by Brewer, Bromley, Gilles, and Lofgren [7] and by Kubaschewski and Evans [8], which are now generally known to be low. The difficulties encountered in the purification of titanium trichloride and its reactivity toward oxygen and moisture have no doubt contributed to the scarcity of experimental data.

We conducted a number of experiments on the reaction between crystalline TiCl_3_ and gaseous chlorine in a calorimetric vessel originally designed for measurement of the heat of formation of boron trichloride [9]. Although TiCl_3_(c) is readily converted to TiCl_4_(g) by the action of chlorine at about 300 °C, a portion of the finely divided sample was carried away by the gas stream. The insertion of a glass wool plug tended to reduce the transfer of TiCl_3_, but the presence of adsorbed moisture introduced significant errors in the determination of the quantity of reaction.

We therefore decided to use a solution calorimeter to make a comparison between the heats of formation of TiCl_3_(c) and TiCl_4_(liq). When TiCl_3_(c) is added to aqueous acid containing an oxidizing agent, the titanium is converted to the tetravalent state; the resulting solution may be reproduced by adding TiCl_4_ to an acid solution which contains the reduced form of the oxidizing agent. The difference between the heats of formation of TiCl_4_ and TiCl_3_ may be determined from a combination of the heat of oxidation and hydrolysis of TiCl_3_ with the heat of hydrolysis of TiCl_4_ and with certain auxiliary data. This method is similar to that employed by Clifton and MacWood [1], except that the experiments were performed at 30 °C instead of 0 °C and iodine was used as the oxidizing agent instead of FeCl_3_.

## 2. Source and Purity of Materials

The TiCl_4_ and TiCl_3_ were prepared by the Inorganic Chemistry Section of the Chemistry Division. The purity of the TiCl_4_ was determined to be 99.99 mole percent by freezing point determinations made in the Pure Substances Section of the Chemistry Division. This material was the same as that used previously for the determination of the heat of formation of TiI_4_ [9].

The purity of the TiCl_3_ was determined by the Inorganic Chemistry Section to be greater than 99.8 percent. Analyses of the solutions obtained after hydrolysis indicated the purity to be 100 ± 0.2 percent.

The iodine, potassium iodide, and hydrochloric acid were reagent-grade materials used with no further purification. The hydriodic acid was redistilled and the constant-boiling fraction collected under a nitrogen atmosphere.

## 3. Apparatus and Procedure

The apparatus was the same as that used for the determination of the heat of formation of TiI_4_ [9], except for minor changes. Capillary inlet and exit tubes were inserted through the calorimeter head to permit removal of oxygen by flushing with nitrogen. Calorimeter temperatures were measured by means of a platinum resistance thermometer. The thermometric system, the apparatus for measurement of electrical energy, and the calorimetric procedure have been described in previous reports [11, 12].

### 3.1. Hydrolysis of TiCl_4_ (liq)

Samples, approximately 0.01 mole each, of the TiCl_4_ were transferred into thin-walled, spherical Pyrex bulbs and sealed in vacuum. The calorimetric solution consisted of 0.01 mole KI, 0.02 mole I_2_, 0.40 mole HC1, 0.01 mole HI, and 25.33 moles water. The iodine and KI were dissolved in a small quantity of water before adding the other materials; the HI was taken from a constant-boiling solution and the HCl from a prepared 4N solution. The remainder of the water was added to bring the total weight up to the previously calculated value. The calorimeter was then assembled, placed in the constant-temperature calorimeter jacket, the stirrer connected, and oxygen removed by flushing with nitrogen. The inlet and exit tubes were then closed, leaving a slight positive pressure of about 1 mm above atmospheric pressure in the calorimeter.

Calorimeter temperatures were noted at 2-minute intervals during the initial rating-period, after which the sample bulb was crushed and temperatures measured at 1-minute intervals until thermal equilibrium was reestablished. Temperatures were then noted at 2-minute intervals during a final rating-period.

The rate of hydrolysis was quite rapid, the hydrolysis being essentially complete within one minute. In a few cases some of the calorimetric solution was splashed upon the upper walls of the vessel; in such cases the experiment was discarded. The reaction produced no noticeable difference in the color of the solution.

The calorimeter was calibrated in the same manner, except that an empty bulb was used and the temperature rise was derived from a carefully measured quantity of electrical energy introduced into the system.

### 3.2. Hydrolysis of TiCl_3_(s)

Samples, approximately 0.01 mole each, of the finely divided TiCl_3_ were transferred into glass bulbs in a dry-box under an atmosphere of nitrogen. The bulbs were capped while in the box, then removed and sealed.

The calorimetric solution was the same as that used for the experiments with TiCl_4_, except that the HI was omitted and the quantities of iodine and HCl were increased to 0.025 and 0.41 mole respectively. The calorimetric procedure was the same as that described for the TiCl_4_ experiments.

The rate of hydrolysis was quite rapid at first, but quickly slowed down; the hydrolysis was not complete after 1½ hrs at 25 °C. We found it necessary to raise the calorimeter temperature to 30 °C in order to obtain complete hydrolysis within one hour. The resulting solutions developed some turbidity after standing in air for a few hours. In a few cases the solutions were stored in completely filled and stoppered amber bottles; in these cases no turbidity appeared even after several days.

The system was calibrated with electrical energy, at 25 and at 30 °C, using both the initial and final solutions in order to obtain Δ*Cp* for the actual calorimetric process. The effective heat capacity of the empty calorimeter was determined by substituting a known weight of distilled water for the calorimetric solution.

### 3.3. Heat of Solution of Iodine

The iodine samples, approximately 0.005 mole each, were sealed into glass bulbs. The calorimetric solution was the same as used for TiCl_3_ experiments, except that only 0.02 mole of iodine was used. Because the solution of iodine resulted in a relatively small drop in the calorimeter temperature, it was possible to calibrate the actual system immediately prior to the measurement of the heat of solution and it was not necessary to duplicate the system exactly for replicate experiments.

### 3.4. Heat of Dilution of Hydrochloric Acid

A solution of hydrochloric acid containing 13.5 moles of water per mole of HCl was prepared. Samples of this solution, each containing approximately 0.01 mole of HC1, were sealed into glass bulbs. The calorimetric solution consisted of 0.10 mole KI, 0.02 mole I_2_, 0.40 mole HCl, and 25.195 moles of water. Since the increase in the calorimeter temperature was smell, it was possible to calibrate the actual system before each experiment as described above.

## 4. Data and Calculations

Values from the 1955 table of International Atomic Weights [13] were used in all calculations. Except where specified to the contrary, the final temperature and pressure were 25 °C and 1 atmosphere. One thermochemical calorie is taken as equivalent to 4.1840 joules.

The results of the calibration experiments on the TiCl_4_ system are given in table 1. *E* is the electrical energy introduced into the system as measured by a method described previously [11, 12]. Δ*Rc* is the temperature rise of the calorimetric system as measured with a particular platinum resistance thermometer and bridge, corrected by a method described previously [14]. The energy equivalent of the system *E_s_*, is the ratio of the quantity of electrical energy to the resulting rise in temperature:

$$E_{s}=-\frac{E}{\Delta Rc}.$$

The results of the experiments on the hydrolysis of T_1_Cl_4_ are given in table 2, where Δ*e* is the deviation in the energy equivalent of the actual system from that of the calibrated system, Δ*Rc* is the temperature rise of the system, and *q* is the total energy evolved in the actual process. The following relationship was used:

$$q=\left(E_{s}+\Delta e\right)\left(\Delta Rc\right).$$

The data given in table 2 for the hydrolysis of TiCl_4_ correspond to the process:

$$\begin{array}{l} {\text{TiCl}}_{4}\left(\text{liq}\right)+\left[10\text{KI}+2I_{2}+\text{HI}+40\text{HCl}+2533H_{2}O\right]\;(\text{soln})\to \\ \left[{\text{Ti}}^{4+}+4{\text{Cl}}^{-}+10\text{KI}+2I_{2}+\text{HI}+40\text{HCl}+2533H_{2}O\right]\;(\text{soln});\\ \Delta H\left(25\;{}^{\circ}C\right)=-57.14\pm 0.18\;\text{kcal}/\text{mole}. \end{array}$$ (1)

The results of the calibration experiments on the TiCl_3_ system and of the oxidation and hydrolysis experiments on TiCl_3_ are given in tables 3 and 4 respectively. These experiments were conducted at 30 °C in order to increase the rate of hydrolysis. Because of the relatively long period of time required for the establishment of thermal equilibrium, we failed to adjust the system precisely to a final temperature of 30 °C. A correction (*q_t_*) for the thermal coefficient of the process was made to correct all data to this temperature. The data given in table 4 correspond to the process:

$$\begin{array}{l} {\text{TlCl}}_{3}\left(c\right)+\left[10\text{KI}+2.5I_{2}+41\text{HCl}+2533H_{2}O\right]\;\left(\text{soln}\right)\to \\ \left[{\text{Ti}}^{4+}+4{\text{Cl}}^{-}+10\text{KI}+2I_{2}+\text{HI}+40\text{HCl}+2533H_{2}O\right]\;\left(\text{soln}\right),\\ \Delta H\left(30\;{}^{\circ}C\right)=-51.87\pm 0.21\;\text{kcal}/\text{mole}. \end{array}$$ (2)

The value of Δ*Cp* for the process corresponding to eq (2) was determined to be −82 cal/°C mole, this gives for eq (2),

ΔH(25°C)=−51.46±0.21kcal/mole.

The results of the experiments on the heat of solution of iodine, given in table 5, correspond to the process:

$$\begin{array}{l} ½I_{2}\left(c\right)+\left[10\text{KI}+2I_{2}+41\text{HCl}+2533H_{2}O\right]\;\left(\text{soln}\right)\to \\ \;\left[10\text{KI}+2.5I_{2}+41\text{HCl}+2533H_{2}O\right]\;\left(\text{soln}\right);\\ \Delta H\left(25\;{}^{\circ}C\right)=1.101\pm 0.035\;\text{kcal}/\text{mole}\;I_{2}. \end{array}$$ (3)

The results of the experiments on the dilution of hydrochloric acid are given in table 6 and correspond to the process:

$$\begin{array}{l} \left[\text{HCl}+13.5H_{2}O\right]\;\left(\text{soln}\right)+\left[10\text{KI}+2I_{2}+40\text{HCl}+2519.5H_{2}O\right]\;\left(\text{soln}\right)\to \\ \;\left[10\text{KI}+2I_{2}+41\text{HCl}+2533H_{2}O\right]\;\left(\text{soln}\right);\\ \Delta H\left(25\;{}^{\circ}C\right)=-0.603\pm 0.004\;\text{kcal}/\text{mole}\;\text{HCl}. \end{array}$$ (4)

The heats of solution of HCl(gas) and of HI (gas) have been calculated from available data [15] for the following processes:

$$\text{HCl}\left(\text{gas}\right)+13.5H_{2}O\left(\text{liq}\right)\to \left[\text{HCl}+13.5H_{2}O\right]\;\left(\text{soln}\right);$$ (5)

$$\begin{array}{l} \;\Delta H\left(25\;{}^{\circ}C\right)=-16.77\pm 0.01\;\text{kcal}/\text{mole},\\ \text{HI}\left(\text{gas}\right)+\left[10\text{KI}+2I_{2}+40\text{HCl}+2533H_{2}O\right]\;\left(\text{soln}\right)\to \\ \;\left[10\text{KI}+2I_{2}+\text{HI}+40\text{HCl}+2533H_{2}O\right]\;\left(\text{soln}\right);\\ \;\Delta H\left(25\;{}^{\circ}C\right)=-19.51\pm 0.10\;\text{kcal}/\text{mole}. \end{array}$$ (6)

The heat involved in diluting the KI–I_2_–HCl solution by approximately 0.5 percent has been calculated to be negligible within the precision of these experiments. For simplification of calculations, however, the following process has been included:

$$\begin{array}{l} 13.5H_{2}O\left(\text{liq}\right)+\left[10\text{KI}+2I_{2}+40\text{HCl}+2519.5H_{2}O\right]\;\left(\text{soln}\right)\\ \;\to \left[10\text{KI}+2I_{2}+40\text{HCl}+2533H_{2}O\right]\;\left(\text{soln}\right);\\ \;\Delta H\left(25\;{}^{\circ}C\right)=0.00\pm 0.01\;\text{kcal}/\text{mole}. \end{array}$$ (7)

We may subtract eqs (1), (6), and (7) from the sum of eqs (2), (3), (4), and (5) to obtain the following theoretical process:

$$\begin{array}{l} {\text{TiCl}}_{3}\left(c\right)+½I_{2}\left(c\right)+\text{HCl}\left(\text{gas}\right)\to {\text{TiCl}}_{4}\left(\text{liq}\right)+\text{HI}\left(\text{gas}\right);\\ \;\Delta H{}^{\circ}\left(25\;{}^{\circ}C\right)=8.37\pm 0.30\;\text{kcal}/\text{mole}. \end{array}$$ (8)

The change in enthalpy for eq (8) may be combined with −22.063±0.002 and 6.20±0.10 kcal/mole, the standard heats of formation of HCl(gas) and HI (gas) [15] respectively, to give:

$$\begin{array}{l} \;{\text{TiCl}}_{3}\left(c\right)+\frac{1}{2}{\text{Cl}}_{2}\left(\text{gas}\right)\to {\text{TiCl}}_{4}\left(\text{liq}\right);\\ \Delta H{}^{\circ}\left(25\;{}^{\circ}C\right)=-19.89\pm 0.32\;\text{kcal}/\text{mole}. \end{array}$$ (9)

By taking −192.3 ±0.7 kcal/mole for the heat of formation of TiCl_4_(liq) [16],^2^ we obtain:

$${\text{TiCl}}_{3}\left(c\right),\Delta \text{Hf}{}^{\circ}\left(25\;{}^{\circ}C\right)=-172.4\pm 0.8\;\text{kcal}/\text{mole}.$$

The results obtained from the experiments on the direct chlorination gave −19.9±1.3 kcal/mole for the process corresponding to eq (9).

The uncertainties assigned to the values given in this paper are over-all uncertainties obtained by combining twice the standard deviation of the mean for the calibration and reaction experiments with reasonable estimates of all other known sources of error.

## 5. Discussion

The value obtained in this investigation is essentially the same as that obtained by Clifton and MacWood [1] from measurements of the energies evolved when TiCl_3_(c) and TiCl_4_(liq) were dissolved in an aqueous solution of HC1 and FeCl_3_.

Schaffer, Bred, and Pfeffer [2] measured the heat of chlorination of TiCl_3_ at 57 °C and obtained −20.3 kcal/mole for the process corresponding to eq (9). They also measured the heat of reduction of TiCl_4_ at 153 °C, with mercury as the reducing agent, and obtained −19.8 kcal/mole for eq (9). The values which they reported for the heat of formation of TiCl_3_, however, were based upon the value calculated by Bichowsky and Rossini [17] for the heat of formation of TiCl_4_(liq), which has since been shown to be low. If we combine the data obtained by Schaffer, Bred, and Pfeffer with −192.3±0.7 kcal/mole for the heat of formation of TiCl_4_(liq), we obtain −172.0 and −172.5 kcal/mole for the heats of formation of TiCl_3_(c) obtained by the chlorination and reduction processes, respectively.

Krieve and Mason [3] studied the equilibrium:

$${\text{TiCl}}_{3}\left(c\right)+\text{HCl}\left(\text{gas}\right)={\text{TiCl}}_{4}\left(\text{gas}\right)+\frac{1}{2}H_{2}\left(\text{gas}\right),$$

and obtained ΔH_690_= 10.4 kcal/mole. They estimated the Δ*Cp* of the process to be −6 cal/deg mole, used Kelley’s value of 9.6 kcal/mole [18] for the heat of vaporization of TiCl_4_(liq) [2], and obtained −19.0 kcal/mole for eq (9). We have corrected their data by the use of 9.9 kcal/mole for the heat of vaporization of liquid TiCl_4_ [2] and have obtained −19.3 kcal/mole for the process corresponding to eq (9). Combination of this value with −192.3 kcal/mole for the heat of formation of TiCl_4_(liq), gives −173.0 kcal/mole for the heat of formation of TiCl_3_(c).

Krieve, Vango, and Mason [4] reacted Ti(c), TiCl_2_(c), and TiCl_3_(c) with chlorine under pressure in a nickel-bomb calorimeter to form TiCl_4_(liq). They obtained −190.0 and −20.9 kcal/mole for the heats of chlorination of Ti(c) and TiCl_3_(c), respectively, and reported −169.1 kcal/mole for the heat of formation of TiCl_3_(c). If we take their value for the heat of chlorination of TiCl_3_(c) and use our value for the heat of formation of TiCl_4_(liq), we obtain −171.4 kcal/mole for the heat of formation of TiCl_3_(c).

Skinner and Ruehrwein [5] measured the heats of solution of Ti(c) and TiCl_3_(c) in aqueous hydrofluroic acid. They obtained −170.0 kcal/mole for the heat of formation of TiCl_3_(c).

Altman, Farber, and Mason [6] have reported values of −169.4 and −171.0 kcal/mole for the heat of formation of TiCl_3_(c), based upon disproportionation and sublimation studies by Farber and Darnell [19, 20].

A summary of the results obtained by the various investigators is given in table 7. An examination of the results in this table indicates substantial agreement on the difference between the heats of formation of TiCl_3_(c) and TiCl_4_(liq).

## Figures and Tables

**Table 1:** Results of the calibration experiments on the *TiCl*_4_ system

Experiment	*E*	Δ*Rc*	*E_s_*
			
	*j*	*ohm*	*j*/*ohm*
1…………..	1939.62	0.090254	21491
2…………..	1937.46	.090022	21522
3…………..	1936.64	.090053	21506
4…………..	1934.99	.090001	21500
5…………..	1936.37	.090235	21459
6…………..	1931.84	.090000	21465
	
Mean			21490
Standard deviation of the mean			±10

**Table 2:** Results of the experiments on the hydrolysis of *TiCl*_4_

Experiment	Δ*e*	Δ*Rc*	*q*	TiCl_4_ mole	−ΔH(25 °C)
					
	*j/ohm*	*ohm*	*j*		*kj*/*mole*
1………….	7.9	0.069850	1501.7	0.0062711	239.46
2………….	9.7	.082721	1778.3	.0074704	238.04
3………….	11.0	.091447	1966.2	.0082202	239.20
4………….	10.2	.085143	1830.6	.0076219	240.18
5………….	12.2	.099699	2143.8	.0089877	238.53
	
Mean					239.08
Standard deviation of the mean					±0.37

**Table 3:** Results of the calibration experiments on the *TiCl*_3_ system

Experiment	*E*	Δ*Rc*	*E_s_*(30° C)
			
	*j*	*ohm*	*j*/*ohm*
1………….	2282.25	0.105484	21636.0
2………….	2420.74	.111916	21630.0
3………….	2447.40	.113123	21634.9
4………….	2407.39	.111278	21634.0
5………….	2406.49	.111193	21642.5
	
Mean			21635.5
Standard deviation of the mean			±2.0

**Table 4:** Results of the *TiCl*_3_ oxidation and hydrolysis experiments

Experiment	Δ*e*	Δ*Rc*	*q*	*q_t_*	TiCl_3_ mole	−ΔH(30 ° C)
						
	*j*/*ohm*	*ohm*	*j*	*j*		*kj*/*mole*
1………..	11.5	0.126488	2738.09	5.59	0.0127267	215.58
2………..	7.8	.089936	1946.51	−0.13	.0089266	218.04
3………..	8.4	.095814	2073.79	−0.26	.0095228	217.74
4………..	8.2	.095842	2074.38	−0.31	.0095531	217.11
5………..	10.1	.109841	2377.57	−0.15	.0109743	216.64
	
Mean						217.02
Standard deviation of the mean						±0.44

**Table 5:** Results of the experiments on the solution of iodine

Experiment	*Es*	*—ΔRc*	*q*	I_2_mole	ΔH (25 ° C)
					
	*j*/*ohm*	*ohm*	*j*		*kj*/*mole*
1……….	21231	0.001051	22.31	0.0050000	4.462
2……….	21463	.000850	18.24	.0041636	4.381
3……….	21438	.000912	19.55	.0044386	4.405
4……….	21605	.001088	23.51	.0050785	4.629
5……….	21454	.001149	24.65	.0050579	4.874
6……….	21468	.001013	21.75	.0048742	4.462
7……….	21574	.001086	23.43	.0049308	4.752
8……….	21535	.001136	24.46	.0049956	4.896
	
Mean					4.608
Standard deviation of the mean					±0.074

**Table 6:** Results of the experiments on the dilution of hydrochloric acid

Experiment	*E_s_*	Δ*Rc*	*q*	HCl mole	−ΔH(25 °C)
					
	*j*/*ohm*	*ohm*	*j*		*kj/mole*
1…………..	21622	0.001109	23.98	0.0094364	2.541
2…………..	21588	.000972	20.98	.0083074	2.525
3…………..	21441	.001158	24.83	.0099317	2.500
4…………..	21425	.001236	26.48	.0104966	2.523
	
Mean					2.522
Standard deviation of the mean					±0.008

**Table 7:** Heat of formation of *TiCl*_3_ reported by the various investigators, kcal/mole at 25 °C

$\Delta {\text{Hf}}_{{\text{TiCl}}_{3}}\left(s\right)-\Delta {\text{Hf}}_{{\text{TiCl}}_{4}}\left(\text{liq}\right)$	$-\Delta {\text{Hf}}_{{\text{TiCl}}_{3}}\left(s\right)$	Reference
		
19.9	172.4	1
20.3	172.0	2
19.8	172.5	2
19.3	173.0	3
20.9	169.1	4
	171.4	4
	170.0	5
	169.4	6
	171.0	6
19.9±0.3	172.4±0.8	This investigation.

## Footnotes

1 Figures in brackets indicate the literature references at the end of this paper.

2 The heat of vaporization of TiCl_4_(liq) has been taken as 9.9 ±0.2 kcal/mole from the calorimetric data of Schaffer, Breil, and Pfeffer [2].
